# First-year growth of 834 preterm infants in a Chinese population: a single-center study

**DOI:** 10.1186/s12887-019-1752-8

**Published:** 2019-11-04

**Authors:** Ying Deng, Fan Yang, Dezhi Mu

**Affiliations:** 1 Department of Pediatrics, West China Second University Hospital, Chengdu, Sichuan Province China; 2 Key Laboratory of Birth Defects and Related Disease of Women and Children, Ministry of Education, Sichuan University, Chengdu, 610041 Sichuan Province China

**Keywords:** Preterm infants, Catch-up growth, Anemia

## Abstract

**Background:**

The aim of this study was to follow the growth and hematological indicators of preterm infants during their first year.

**Methods:**

Neonates below 37 gestational weeks had routine follow-ups up through 1 year from January 2012 to December 2015 at West China 2nd University Hospital, Sichuan University. Weight, length and head circumference (HC) were measured monthly during the first 6 months, followed by monitoring every second month until 12 months. The catch-up growth defined as a gain of Z-score > 0.67 according to previous study. All preterm infants were prescribed iron prophylaxis based on national guideline. The hemoglobin concentration was examined at 6 and 12 months.

**Results:**

Altogether, 132 very-low-birth-weight (VLBW), 504 low-birth-weight (LBW) and 198 normal-birth-weight (NBW) infants were followed. The rates of catch-up growth for weight, length and HC 12 months of corrected age (CA) were 22.6, 29.1 and 14.6%, respectively. SGA and VLBW infants showed higher catch-up growth rates. The overall prevalence of anemia was 6.8% at 6 months and 7.8% at 12 months. The Z-scores for weight-for-length, length and HC were lower in the VLBW and SGA preterm infant groups than in the other preterm groups throughout the first year of life. The incidences of stunting, microcephaly and wasting changed from 5, 1.3 and 3.7% to 2, 1.1, 0.9 and 2.4%, respectively, during the first year. However, the incidences of wasting and stunting were higher for the VLBW infants than for the LBW and NBW infants at 12 months (9.3% vs. 1.4%, *p* < 0.01; 9.3% vs. 1%, *p* < 0.01,respectively; 4.7% vs. 0.8%, *p* < 0.01, 4.7% vs. 0%, *p* < 0.01,respectively). Similar results were observed between SGA and AGA infants (8.7% vs. 1.5%, *p* < 0.01; 5.8% vs. 0.4%, *p* < 0.01). Logistic regression revealed SGA and VLBW as risk factors for poor growth (WLZ < -2SD) at 12 months (OR = 5.5, 95% CI: 2.1–14.8, p < 0.01: OR = 4.8, 95% CI: 1.8–12.8, *p* < 0.01, respectively).

**Conclusion:**

The VLBW and SGA preterm infants showed significant catch-up growth during their first year of life. However, SGA and VLBW were risk factors for poor growth during the preterm infants’ first year of life. Prophylactic iron supplementation in preterm infants appears to reduce the prevalence of anemia.

## Background

Approximately 11.1% of live births worldwide are preterm, with an estimated rate ranging from 7.2 to 13% in Asia [1]. Due to dramatic advances in perinatal care, preterm infants, including very-low-birth-weight (VLBW) preterm infants (birth weight < 1500 g), are able to survive the first few weeks of life. Because survival is an expected outcome, the focus has shifted toward improving nutrition and anthropometry. However, VLBW and small-for-gestational age (SGA) preterm infants have a higher risk of growth deviations [2]. Several studies have shown an association between impaired extra-uterine growth and poor long-term performance [3]. The catch-up growth patterns of preterm infants have been a matter of debate. Zhao et al. [4] observed no catch-up growth in weight and length among preterm infants, while Westerberg et al. [5] observed that VLBW infants showed significant catch-up growth in both weight and length during the first year of life. Thus, an understanding of the early growth patterns of preterm infants is important and may help improve appropriate daily care practices and reduce the morbidity of growth deviations related to preterm birth.

Growth is often assessed by comparing the weight, length, and head circumference (HC) of a child with growth references to a given society. Until now, no general growth references for preterm infants have existed. Fenton’s growth chart considers preterm infants, but their data are relevant only for the first 40 weeks of the postnatal period, and the chart uses data from cross-sectional studies and thus cannot provide longitudinal growth trajectories with specific gestational ages. Longer term assessments must rely on the growth chart developed by the World Health Organization (WHO). In 2006, the WHO developed growth standards for full-term infants. To follow the longer term growth of preterm infants, the WHO growth chart is appropriate beginning at 40 weeks (0 month) of corrected age (CA). Most investigators choose the WHO growth chart to assess the growth of preterm infants from 40 weeks CA [5–8].

Anemia is defined as an Hb level two standard deviations below the mean for the normal population when matched for age and sex. Anemia has become a major worldwide public health problem that affects up to 50% of preschool-aged children. In China, the overall rate of pediatric anemia is 19% (95% CI: 6–38%) [9]. Anemia is most often caused by iron deficiency. Iron is an essential nutrient and plays an important role in multiple processes, including pediatric growth and development [10]. Premature infants are particularly vulnerable to the development of anemia due to lower storage concentrations of iron, shorter red-blood-cell half-lives and a greater need for iron. A few reports have shown that the prevalence of iron deficiency is estimated to range between 25 and 80% among preterm infants during infancy [11]. The overall rate of infant and childhood anemia is as low as 6% in developed countries [10]. However, the prevalence of anemia remains high in low-income countries [8]. Ferri et al. [12] reported a high incidence of both iron-deficiency anemia (26.5%) and iron deficiency (48%) in a Brazilian preterm VLBW population during infancy. The prevalence of iron deficiency anemia in term infants was 20.8% for 7- to 12-month-old infants in China in 2000 [13]. However, little is known regarding the prevalence of anemia in preterm infants in China.

Few cohort studies have described the first year growth and hematologic indicators of preterm infants. The aim of this study was to examine the Z-scores of weight, length and HC and the hemoglobin concentration level during the first year of life in a Chinese population cohort.

## Materials and methods

### Study population

**Fig. 1:**
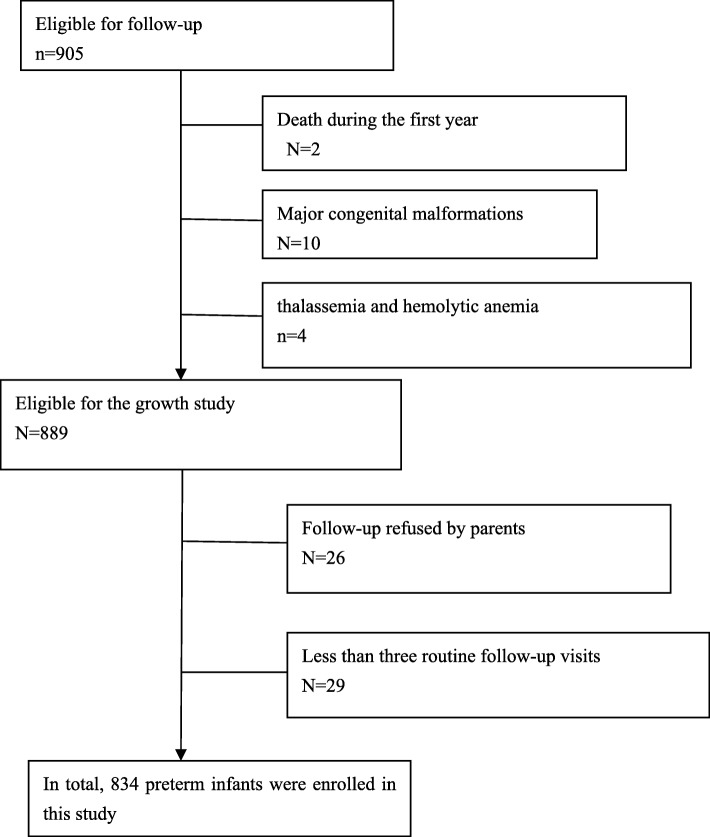
study population

This longitudinal study comprised all infants born before 37 complete gestational weeks between January 2012 and December 2015 who were admitted to the pediatric health department of the Second University Hospital of Sichuan University. These infants had been followed up to 12 months of age. The exclusion criteria were a gestational age (GA) > 37 weeks, major congenital malformations, chromosomal abnormalities or genetic syndromes, thalassemia or hemolytic anemia and failure to attend more than three routine follow-up visits. GA was determined based on the mother’s last menstrual period or first-trimester ultrasonogram. In total, 834 preterm infants were included in this study (Fig. 1). Ethical approval was granted from the ethics committee at the West China Second University Hospital, and informed consent was obtained from all parents or guardians before the study(Chuan201021).

### Data collection

#### Anthropometric measurements

Preterm infant growth was monitored monthly for weight, length and HC during the first 6 months, followed by monitoring every second month until 12 months. Month 0 CA was the first time point which growth was evaluated. Growth data were obtained at the end of each month of age (±3 days) by trained nurses at the Department of Child Health Care of the West China Second University Hospital. Infants were weighed unclothed using digital infant scales (HJ-3000, HuaJu, Zhe Jiang,China), and the weight accuracy was within 0.01 kg. Lengths were measured by an infant length board (WS-TCS-20, Sheng Yuang, Shanghai) and recorded to the nearest 0.1 cm. HC was measured with a flexible non-stretch tape and recorded to the nearest 0.1 cm.

#### Hematological indicators

Blood tests were performed to screen for anemia at 6 and 12 months. The blood collection method were as follows: 1) disinfect the ring finger with alcohol-covered cotton balls; 2) puncture it with a disposable blood needle and let the blood naturally flow out; and 3. after the first drop was lightly wiped with a sterile cotton ball, the blood was collected in a 0.5-ml centrifuge tube (EDTAK2 anticoagulant tube). The sample was approximately 0.2–0.3 ml. A Sysmex automated blood analyzer-XT-1800i (Japan) was used to detect Hb concentration, mean corpuscular Hb concentration, and mean corpuscular volume. At 12 months, anemia was diagnosed based on the presence of Hb levels< 110 g/L, and at 6 months, anemia was diagnosed based on the presence of Hb levels< 100 g/L [14].

#### Feeding recommendations

All preterm infants were prescribed iron prophylaxis (3–4 mg/kg/day) and vitamin D (800 IU /day before 3 months and 400 IU /day thereafter), and treatments were continued throughout the first year of life with dosage adjustments according to physical growth conditions. The feeding recommendations were as follows: 1, low-risk preterm infants (BW > 2000 g, GA > 34 weeks), breastfeeding or formula feeding; 2, moderate risk (1500 g ≤ BW ≤ 2000 g, 32 weeks≤GA ≤ 34 weeks), supplement breastfeeding with fortified breastmilk or post-discharge formula for up to 3–6 months of CA; and 3, high risk (BW < 1500 g, GA < 32 weeks), supplement breastfeeding with fortified breastmilk or post-discharge formula through 6–12 months of CA [15].

#### Statistical analysis

Weight, length and HC were converted to Z-scores for gender and the CA based on the WHO Child Growth Standards [WHO Anthro (version 3.2.2, January 2011), https://www.who.int/childgrowth/software/en/]. The CA was calculated by subtracting the difference between term birth and the GA at birth from the child’s chronological age. Anthropometric data are presented as Z-scores based on the WHO growth charts. Z-score means were calculated as follows: (observed value minus the median value of the reference population) /standard deviation (SD). SGA was defined as a birth weight below the 10th percentile for GA based on Fenton’s growth chart for 22–50 weeks [16]. Stunting was defined as a length-for-age Z-score (LAZ) < − 2 SD; microcephaly was defined as an HC-for-age Z-score (HCZ) < − 2 SD; and wasting was defined as a weight-for-length Z-score < − 2 SD. Overweight was defined as a BMI-for-age Z-score > 2 SD. Poor growth was defined as WLZ < -2SD. Catch-up growth was defined as accelerated growth rate. In the Westerberg.et al’study, they investigated a change in the Z-score > 0.67 between two time points was a cut-off value for catch-up growth [5].

Data analysis was performed using SPSS software version 20.0 for Windows (SPSS Inc., Chicago, IL, USA). Categorical variables (incidence of microcephaly, stunting and wasting, and catch-up growth) were analyzed using the chi-square test or Fisher’s exact test (T < 5). Continuous variables were analyzed using Student’s t-test or ANOVA.

A univariate analysis was performed to identify risk factors for poor growth. Factors were selected a priori after a review of the literature [5, 17]. Variables associated with poor growth at *p* < 0.10 based on the univariate analysis were included in the final logistic regression in a forward, stepwise manner to identify risk factors (SGA, VLBW, gender, gestational age and type of pregnancy). Given that SGA and VLBW are highly correlated variables, we constructed two models including either SGA or VLBW. Model 1 includes SGA, gender, gestational age and type of pregnancy. Model 2 includes gender, gestational age, VLBW, and type of pregnancy [18]. GraphPad Prism (version 6.02, San Diego, California, USA) was used to plot growth Z-score curves at different ages, which were fitted by a third-order polynomial model. *P* values < 0.01 were considered statistically significant.

## Results

A total of 834 preterm infants with a mean GA of 34.2 ± 2.3 (range 26–36.9) weeks and a mean birth weight (BW) of 2108.5 ± 536.0 (range 820–3750) grams were enrolled in this study. The study included a total of 103 SGA infants; 27 (20.9%) were VLBW infants, and 76 (15.1%) were low-birth-weight (LBW) infants. The baseline characteristics of the study participants are summarized in Table 1.

**Table 1:** Baseline characteristics of preterm infants (*n* = 834)

Characteristics	VLBW	LBW	NBW
Number of participants	132	504	198
Gestational age (week)	30.6 ± 2	34.5 ± 1.6	36.0 ± 0.8
Birth weight (kg)	1.27 ± 0.16	2.06 ± 0.28	2.79 ± 0.23
WAZ for birth	−0.55 ± 1.02	−0.58 ± 0.75	0.27 ± 0.61
Male	61(46)	264(47)	129(65)
Twins	37(28)	194(38)	44(23)
SGA	27(20.9)	76(15.1)	0(0)
Characteristics of mothers			
Education(college)	56(43.1)	249(49.5)	87(44.1)
Primipara	53(40.6)	234(46.5)	109(55.3)
Maternal age at birth (years)	27.0 ± 3.3	27.3 ± 4.1	26.9 ± 3.0

Values are mean ± SD or n (%). WAZ: weight-for-age Z-score; SGA: Small-for-gestational age

### Growth evaluated by Z-scores

**Fig. 2:**
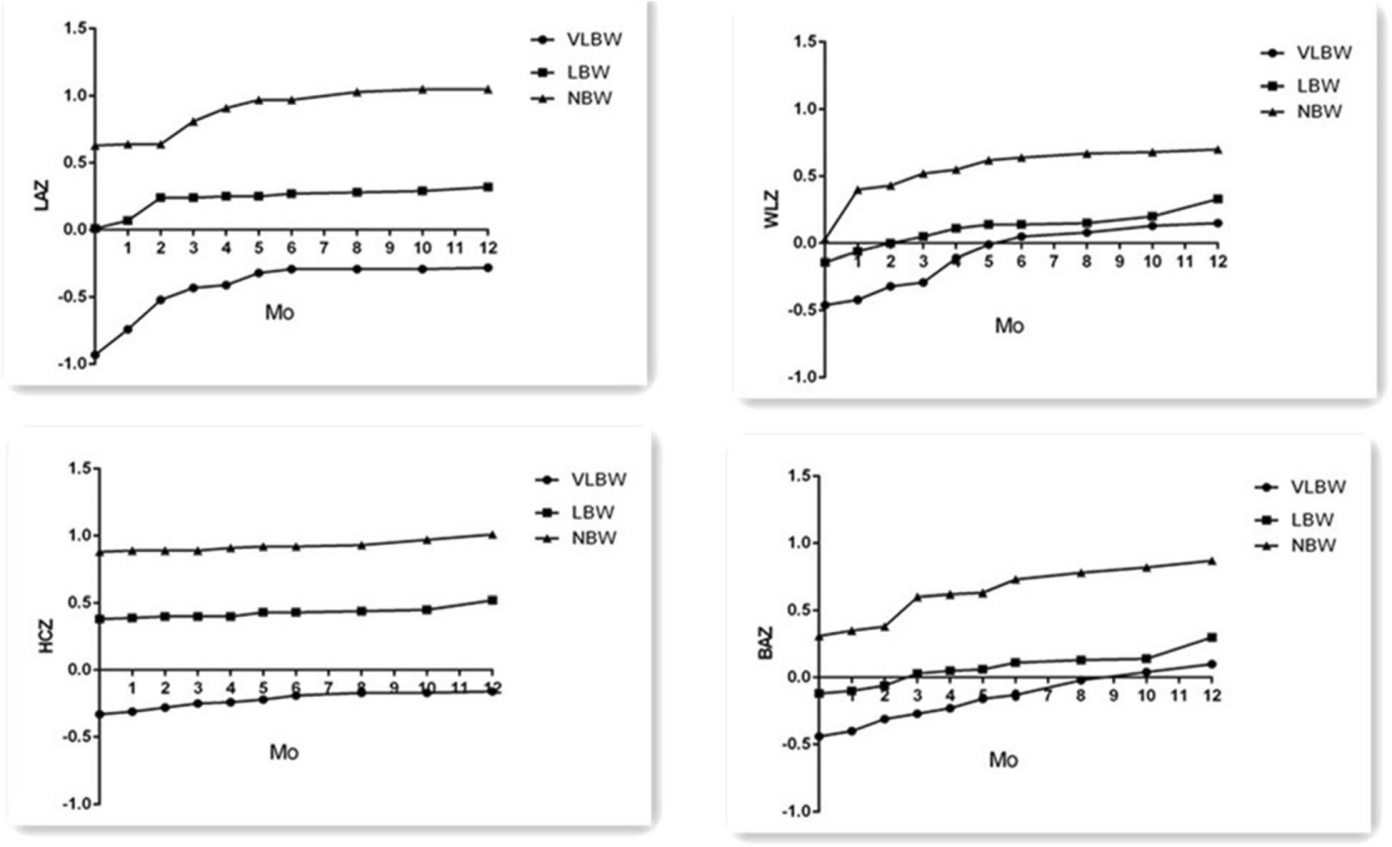
The trajectories of LAZ,WLZ, HCZ and BAZ in different birth weight groups during the first 12 months of corrected age. (**a**) The Z-scores for length for age (LAZ) were significant higher for the normal-birth-weight (NBW) infants than for the low-birth-weight (LBW) and very-low-birth-weight(VLBW) infants, *p* < 0.01. (**b**) The Z-scores for weight for length (WLZ) were significant higher for the NBW infants than for the LBW and VLBW infants, *p* < 0.01. (**c**) The Z-scores for head-circumference for age (HCZ) were significant higher for NBW infants than for the LBW and VLBW infants, *p* < 0.01. (**d**) the Z-scores for body mass index for age (BAZ) were significant higher for NBW infants than for the LBW and VLBW infants, *p* < 0.01

**Fig. 3:**
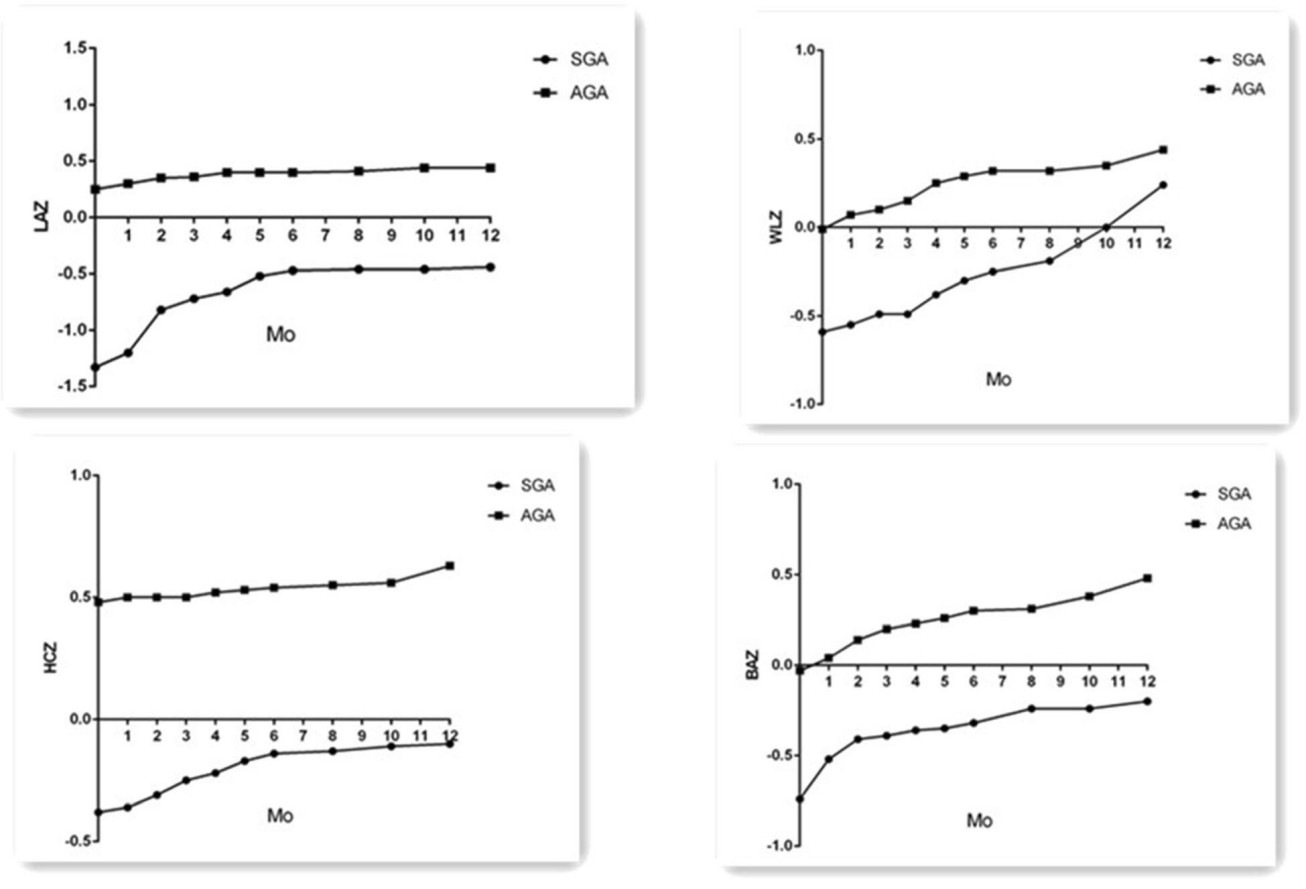
The trajectories of LAZ, WLZ, HCZ and BAZ in different gestational age(GA) preterm infants during the first 12 months of corrected age. (**a**) The Z-scores for length for age (LAZ) were significant higher for appropriate-for-gestational age (AGA) infants than for the Small-for-gestational age (SGA) infants, *p* < 0.01; (**b**) The Z-scores for weight for length (WLZ) were significant higher for AGA infants than for the SGA infants, p < 0.01.(C) The Z-scores for head-circumference for age (HCZ) were significant higher for AGA infants than for the SGA infants, p < 0.01;(D) the Z-scores for body mass index for age (BAZ) were significant higher for AGA infants than for the SGA infants, p < 0.01

Significant differences were evident in the mean LAZ, WLZ, HCZ and BAZ from 0 months of CA to 12 months of CA in the different BW and GA groups (Figs. 2 and 3). These scores were lower for the VLBW infants than for the LBW and NBW infants. Similar results were observed between the SGA and AGA groups. The incidences of stunting, microcephaly and wasting changed from 5, 1.3 and 3.7% to 2, 1.1, 0.9 and 2.4%, respectively, during the first year for the entire cohort. However, the incidences of stunting were higher among the VLBW infants than among the LBW and NBW infants (19.3% vs. 3.4% vs. 0%, *p* < 0.01) at 0 months of CA. The incidences of wasting and stunting were higher for the VLBW infants than for the LBW and NBW infants at 12 months (9.3% vs. 1.4%, *p* < 0.01; 9.3% vs. 1%, *p* < 0.01,respectively; 4.7% vs. 0.8%, *p* < 0.01, 4.7% vs. 0%, *p* < 0.01,respectively) (Table 2). The incidences of stunting, wasting and microcephaly were higher in the SGA group than in the AGA group at 0 and 12 months of CA (Table 3).

**Table 2:** the rates of growth deviations in different BW groups

	N(%)	VLBW N(%)	LBW N(%)	NBW N(%)
0 Mon CA	761	114	472	175
Growth deviations				
Stunting	38(5)	22(19.3)^ab^	16(3.4)^ac^	0(0)^bc^
Wasting	28(3.7)	4(3.6)	20(4.2)	4(6.5)
Microcephalus	10(1.3)	8(7)^ab^	2(0.4)^a^	0(0)^b^
Overweight	36(4.7)	8(7)	17(3.6)	11(6.3)
12 Mon CA	798	107	494	197
Growth deviations				
Stunting	9(1.1)	5(4.7)^ab^	4(0.8)^a^	0(0)^b^
Wasting	19(2.4)	10(9.3)^ab^	7(1.4)^a^	2(1)^b^
Microcephalus	7(0.9)	4(3.7)^a^	2(0.4)^a^	1(0.5)
Overweight	9(1.1)	1(0.9)	3(0.6)	5(2.5)

A: significant difference between the VLBW and LBW groups, *p* < 0.01

B: significant difference the VLBW and NBW groups, *p* < 0.01

C: significant difference the LBW and NBW groups, *p* < 0.01

Mon; months. CA: corrected age; BW: birth weight; VLBW:very low birth weight, birth weight < 1500 g; LBW: low birth weight, 1500 g ≤ birth weight < 2500 g; NBW:normal birth weight,2500 ≤ birth weight<4000 g. Chi-square test or Fisher’s exact test was used to analyze

**Table 3:** the rates of growth deviations the different GA groups

	SGA No.(%)	AGA No.(%)	*p*
0 Mon CA			
N	92	659	
Growth deviations			
Stunting	26(28.3)	12(1.8)	< 0.01*
Wasting	9(10)	19(2.9)	< 0.01*
Microcephalus	4(4.3)	6(0.9)	< 0.01*
Overweight	1(1.1)	31(4.7)	0.108
12 Mon CA			
N	103	686	
Growth deviations			
Stunting	6(5.8)	3(0.4)	< 0.01*
Wasting	9(8.7)	10(1.5)	< 0.01*
Microcephalus	3(2.9)	3(0.4)	< 0.01*
Overweight	0(0)	9(1.3)	0.24

Mon:months, CA: corrected age; GA: gestational age; SGA, Small-for-gestational age: a birth weight below the 10th percentile for gestational age based on Fenton’s growth chart. AGA: appropriate-for-gestational age: a birth weight between the 10th percentile and 90th percentile for gestational age. Chi-square test or Fisher’s exact test was used to analyze. *: statistically significant

### Catch-up growth evaluated by Z-score changes

Catch-up growth in weight, length and HC was observed in this study population. The rates of catch-up growth for weight, length and HC between 0 and 6 months of CA and between 0 and 12 months of CA were 26.4, 28.4 and 12.5%, respectively and 22.6, 29.1 and 14.6%, respectively (Table 4). The rates of catch-up growth for weight, length and HC were higher in the VLBW group than in the LBW and NBW groups at 12 months of CA (35.1% vs. 22.2% vs. 16.9%; 48.9% vs. 30.1% vs. 15.2%; 29.8% vs. 14.1% vs. 7.5%) (Table 5). The rates of catch-up growth for weight, length and HC were higher in the SGA group than in the AGA group at 6 and 12 months of CA (Table 6).

**Table 4:** Rate of catch-up growth at different times (%, n/N)

Catch-up growth	0~6 Mon	0~12 Mon
Weight	26.4 (187/707)	22.6 (162/717)
Length	28.4(201/707)	29.1(209/717)
HC	12.5(88/707)	14.6(105/717)

Mon: months; HC: head circumference; catch-up growth: a change in the Z-score > 0.67 between two time points

**Table 5:** Rate of catch-up growth in the different BW groups (%, n/N)

	VLBW	LBW	NBW
Weight			
0–6 Mon	29(29/100)	26.5(117/441)	24.7(41/166)
0–12 Mon	35.1(33/94) ^ab^	22.2(100/451) ^a^	16.9(29/172) ^b^
Length			
0–6 Mon	50(50/100) ^ab^	28(123/441) ^ac^	16.9(28/166) ^b^
0–12 Mon ac	48.9(46/94) ^ab^	30.1(136/451)	15.7(27/172) ^bc^
HC			
0–6 Mon	19(19/100) ^b^	13.4(59/441) ^c^	6(10/166) ^bc^
0–12 Mon	29.8(28/94) ^ab^	14.1(64/451) ^ac^	7.5(13/172) ^bc^

A: significant difference between the VLBW and LBW groups, *p* < 0.01

B: significant difference between the VLBW and NBW groups, *p* < 0.01

C: significant difference between the LBW and NBW groups, *p* < 0.01

Mon: months; HC: head circumference; VLBW:very low birth weight, birth weight < 1500 g; LBW: low birth weight, 1500 g ≤ birth weight < 2500 g; NBW:normal birth weight,2500 ≤ birth weight<4000 g. Catch-up growth: a change in the Z-score > 0.67 between two time points. Chi-square test or Fisher’s exact test was used to analyze

**Table 6:** Rate of catch-up growth in the different GA groups (%, n/N)

	SGA	AGA	*p*
Weight			
0–6 Mon	47.7(42/88)	23.6(144/610)	< 0.01*
0–12 Mon	43.3(39/90)	19.6(122/623)	< 0.01*
Length			
0–6 Mon	54.5(48/88)	24.9(152/610)	< 0.01*
0–12 Mon	55.5(50/90)	23.4(158/623)	< 0.01*
HC			
0–6 Mon	28.4(25/88)	10.3(63/610)	< 0.01*
0–12 Mon	33.3(30/90)	12(75/623)	< 0.01*

Mon: Months; GA: gestational age HC: head circumference; SGA, Small-for-gestational age (birth weight below 10th); AGA: appropriate-for-gestational age (birth weight between 10th and 90th). Catch-up growth: a change in the Z-score > 0.67 between two time points. *: statistically significant

### Risk factors for poor growth at 12 months

At 12 months of CA, the incidence of poor growth (WLZ < -2SD) was 2.4% (19/798), the level of birth weight in the poor growth group was lower than that in the normal growth group, and the rates of SGA and VLBW were higher in the poor growth group than in the normal growth group (1.65 ± 0.55 kg vs. 2.15 ± 0.51 kg, *p* < 0.0001; 47% vs. 13%, *p* < 0.0001; 52.6% vs. 13.5%, *p* < 0.0001, respectively) (Table 7). The multivariable logistic regression showed SGA and VLBW as risk factors for poor growth (OR = 5.5, 95% CI: 2.1–14.8, *p* < 0.01: OR = 4.8, 95% CI: 1.8–12.8, *p* < 0.01, respectively).

**Table 7:** Clinical characteristics of poor growth at 12 months of CA

	Poor growth	Normal growth	*p*
n	19	779	
BW(kg)	1.65 ± 0.55	2.15 ± 0.51	< 0.0001*
GA (weeks)	33.3 ± 2.9	34.4 ± 2.1	0.22
SGA (n, %)	9(47)	102(13)	< 0.0001*
gender, male (n, %)	13(68.4)	424(54.4)	0.226
Twins (n, %)	6(31.6)	260(33.4)	0.87
VLBW (n, %)	10(52.6)	105(13.5)	< 0.0001*

CA: corrected age; SGA, Small-for-gestational age, birth weight below 10th; VLBW: very low birth weight, birth weight < 1500 g; BW: birth weight; GA: gestational age; Poor growth:WLZ < -2SD. *: statistically significant

### The level of hematological indicators among preterm infants

**Fig. 4:**
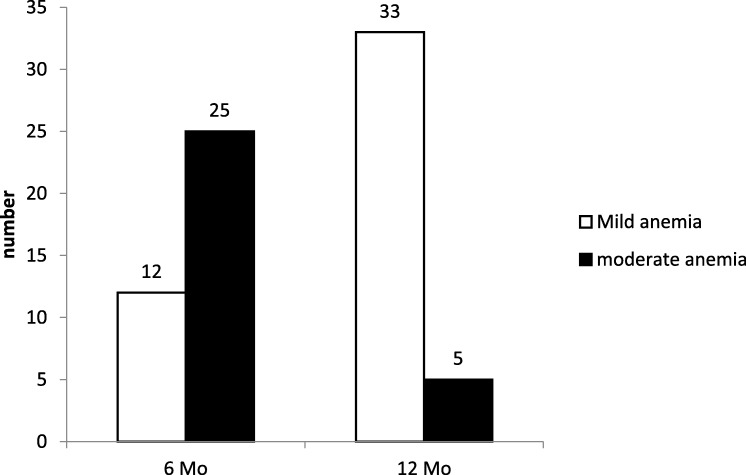
The distribution of varied degrees of anemia in preterm infants. The majority were moderate anemia at 6 months but mild anemia at 12 months in those children with anemia. Mild anemia: 90 g/L ≤ Hb < 100 g/L(6 months) or 110(12 months) g/L. Moderate anemia: 60 g/L ≤ Hb < 90 g/L

Follow-ups for routine blood examinations were available for 546 infants at 6 months and for 485 infants at 12 months. The median Hb concentrations were 119 g/l and 123 g/l at 6 and 12 months of CA, respectively. The overall prevalence of anemia was 6.8% (37/546, 95% CI: 5–9%) at 6 months and 7.8% (*n* = 38/485, 95% CI: 5–10%) at 12 months (Table 8). At 6 months, the incidence rates were 5.6, 7.4, and 5.9% in the VLBW, LBW and NBW groups, respectively (*p* > 0.05). At 12 months, these values were 6.5, 6.5, and 12.3% (p > 0.05) (Tables 9 and 10). Among the children with anemia, the majority had moderate anemia (60 g/L ≤ Hb < 90 g/L) at 6 months but mild anemia at 12 months (90 g/L ≤ Hb < 110 g/L) (Fig. 4).

**Table 8:** Values of routine blood examination [median (p25, p75)]

Age	Hb(g/L)	MCV(fl)	MCH(pg)	MCHC(g/L)	anemia(%,n)
6 Mo	119(116,126)	74.8(72.1,77.2)	26(24.6,27.1)	345(338,352)	6.8(37/546)
12 Mo	123(117,129)	76.9(74.1,79.5)	26.5(25.5,27.4)	343(335,350)	7.8(38/485)

Hb: hemoglobin; MCV: Mean Corpuscular Volume; MCH: Mean Corpuscular Hemoglobin; MCHC: Mean Corpuscular Hemoglobin Concentration. anemia: Hb < 100 g/L at 6 months or Hb < 110 g/L at 12 months

**Table 9:** Rate of anemia in different BW groups[n/N (%)]

Age	VLBW	LBW	NBW	*p*
6 Mon	5/89(5.6)	25/339(7.4)	7/118(5.9)	0.773
12 Mon	4/62(6.5)	20/309(6.5)	14/114(12.3)	0.130

Mon: months. BW: birth weight; VLBW: very low birth weight, birth weight < 1500 g; LBW: low birth weight, 1500 g ≤ birth weight < 2500 g; NBW:normal birth weight,2500 ≤ birth weight<4000 g Anemia: Hb < 100 g/L at 6 months or Hb < 110 g/L at 12 months

**Table 10:** Rate of anemia in different GA groups [n/N (%)]

Age	SGA	AGA	*p*
6 Mon	4/67(6)	33/473(7)	0.760
12 Mon	3/60(5)	35/419(8.4)	0.369

Mon: months; GA: gestational age; SGA, Small-for-gestational age (birth weight below 10th); AGA: appropriate-for-gestational age (birth weight between 10th and 90th)

## Discussion

In this population-based study, we provide continuous data on the post-discharge growth of preterm infants during early infancy. We found that SGA and VLBW were independently associated with lower WLZ, LAZ and HCZ. Correspondingly, the incidences of stunting and microcephaly were higher in the VLBW and SGA preterm infant groups. Previous studies have shown that the growth failure rate among VLBW infants is as high as 50% at 12 months of CA [17, 19, 20]. For SGA preterm infants, Knops et al. [21] found that very preterm AGA infants showed no stunting at 10 years of age; however, many SGA children showed persistent stunting. Nagasaka et al. [22] found that the incidence of short stature was 2-fold higher among preterm infants than among term infants at 3 years of age, but the incidence of short stature was 4.5-fold higher among SGA preterm infants than among term infants. Another study observed that growth failure rates were higher for SGA infants than for AGA infants [20]. Zhao et al. [23] showed that SGA was associated with lower weight and length. The incidence of growth failure in our report was lower than that reported in developing countries [17, 19, 20]. VLBW and SGA preterm infants benefit from improvements in nutritional support and routine follow-up. Although VLBW and SGA preterm infants have a higher risk of poor growth, the rate of poor growth was lower in the present study than in previous reports [17, 19–22].

Our results indicated that SGA preterm infants were prone to accelerated catch-up growth and persistently lower Z-scores. Similar results were obtained for VLBW preterm infants [5, 8, 20]. The peak catch-up growth rates for weight, length and HC occur at 0–6 months of CA, which has important clinical implications. Evidence has shown that growth patterns can influence long-term health [24]. Numerous observational studies have revealed an association between better growth and better long-term neurodevelopmental outcomes in infants born preterm [25, 26]. One study showed that preterm infants who consumed a high-protein formula had a higher IQ and better brain structure in later life than preterm infants who did not consume this formula [6]. Another observational study including 234 moderate and late preterm children showed that poor growth during the first 7 years was associated with poor neuropsychological function [7]. This study showed that the SGA condition with catch-up growth was associated with a significantly higher DQ/IQ from 2 to 4 years of age [8]. In contrast to the neurodevelopmental benefits, the long-term follow-up of preterm infants suggested that faster postnatal weight gain could increase the risk of future metabolic syndromes, especially in SGA infants [26, 27]. Correspondingly, data show a causal link between slower infant growth and a lower risk of later obesity [24, 28]. In contrast, a longitudinal study that enrolled 152 children showed that rapid weight gain in early infancy did not impact metabolic status during adolescence, but rapid weight gain during childhood had an effect on the metabolic status during adolescence [28]. Lifestyle factors during childhood and adolescence have greater impacts on metabolic disease than do early growth and nutritional exposures [29]. According to the present evidence, both poor growth and excessively rapid growth have adverse effects on long-term health. Thus, achieving proper growth in preterm infants is important. Significant catch-up growth in weight, length and HC was observed in preterm infants in our study. Although there is controversy about catch-up growth, the investigators believe that the catch-up growth of preterm infants was substantially improved through careful observation and aggressive interventions [8]. The outcomes showed that the rate of catch-up growth in SGA and VLBW infants was higher than other groups. This may be because the catch up may represent the infants reaching their genetic predisposition for growth. When the unfavorable factors are eliminated, they have the potential for catch-up growth.

In this study, the prevalence of anemia (6.8% at 6 months of CA and 7.8% at 12 months of CA) was relatively low compared with that reported in a review of 7- to 12-month-old infants in China (20.8%) [13]. This discrepancy may be explained by the routine follow-up program available to these preterm infants in our hospital, which provided persistent nutritional support after hospital discharge. The nutritional supports in our study included fortified breastmilk or post-discharge (transitional) formula to ensure adequate weight gain. Furthermore, regular iron, vitamin A and vitamin D supplementation were available and recommended throughout the first year of life. Although anemia may result from various causes, iron deficiency underlies approximately half of cases. In our study, all preterm infants were prescribed iron prophylaxis (3–4 mg/kg/day) and vitamin D (800 IU/day before 3 months and 400 IU/day thereafter). A meta-analysis showed that iron supplementation increases the hematological indicators or iron status level and reduces the rate of anemia or iron deficiency in preterm infants [30]. A study by Lundstrom et al. [31] showed that LBW infants who received iron supplementation (2 mg of iron/kg/day starting at 2 weeks of age) had a lower tendency to develop iron deficiency. Another study by Sherry showed that iron deficiency anemia was decreased due to the usage of enriched breakfast cereal and infant formula [32]. The European Society for Paediatric Gastroenterology, Hepatology, and Nutrition Committee on Nutrition recommends that prophylactic enteral iron supplementation should be started at 2 to 6 weeks of age [33]. The American Academy of Pediatrics recommends that human milk-fed preterm infants should receive a supplement of elemental iron at 2 mg/kg/day starting by 1 month of age for up to 12 months of age, and a preterm infant who is fed formula milk may need an additional iron supplement to reduce the occurrence of anemia, especially iron-deficiency anemia [14].

The main strength of our study is the detailed information on the physical growth and Hb level throughout the first year of life in a large cohort of preterm infants in China. Moreover, we used the WHO growth chart to compute Z-scores. The conversion of anthropometric values to Z-scores involves adjustment for sex and CA. We have described the prevalence of anemia in preterm infants in a population in China, where such data are limited. However, our study has several limitations. Our follow-up period was not long enough. Future investigations should examine the long-term outcomes of preterm infants and specifically examine the influence of early catch-up growth on adulthood. Our study population was sampled from only one province, and the results may not be generalizable to other regions of China. The iron status was not approximately the same in children diagnosed with anemia and iron-deficiency anemia.

## Conclusions

The VLBW and SGA preterm infants exhibited relatively poor growth during the first year of life. However, significant catch-up growth in weight, length and HC was observed. Prophylactic iron supplementation in preterm infants appears to reduce the prevalence of anemia. Our study supports the provision of early-life nutritional care to preterm infants and provides detailed growth information for this population. In addition, the later outcomes of early catch-up growth of preterm infants should be further investigated as the follow-up duration in this population was relatively short.

## Data Availability

The study did not contain confidential patient data. All the data is contained within the manuscript which supports the findings.

## Abbreviations

AGA: Appropriate-for-gestational age
BAZ: BMI-for-age Z-score
BW: Birth weight
CA: Corrected age
Hb: Hemoglobin
HC: Head circumference;
HCZ: HC-for-age Z-score.
LAZ: Length-for-age Z-score
LBW: Low birth weight
MCH: Mean Corpuscular Hemoglobin
MCHC: Mean Corpuscular Hemoglobin Concentration.
MCV: Mean Corpuscular Volume
NBW: Normal birth weight
SGA: Small-for-gestational age: a birth weight below the 10th percentile for gestational age.
VLBW: Very low birth weight
